# Disclosing the Functional Potency of Three Oxygenated Monoterpenes in Combating Microbial Pathogenesis: From Targeting Virulence Factors to Chicken Meat Preservation

**DOI:** 10.3390/foods13060965

**Published:** 2024-03-21

**Authors:** Sarra Akermi, Moufida Chaari, Khaoula Elhadef, Mariam Fourati, Ahlem Chakchouk Mtibaa, Sofia Agriopoulou, Slim Smaoui, Lotfi Mellouli

**Affiliations:** 1 Laboratory of Microbial and Enzymatic Biotechnologies and Biomolecules, Center of Biotechnology of Sfax (CBS), University of Sfax, Road of Sidi Mansour Km 6, P.O. Box 1177, Sfax 3018, Tunisia; sarahakermi221@gmail.com (S.A.); moufida.chaari97@gmail.com (M.C.); elhadefkhawla@gmail.com (K.E.); mariamfourati@ymail.com (M.F.); ahlemchakchouk@yahoo.fr (A.C.M.); lotfi.mallouli@cbs.rnrt.tn (L.M.); 2 Department of Food Science and Technology, University of the Peloponnese, Antikalamos, 24100 Kalamata, Greece; s.agriopoulou@uop.gr

**Keywords:** oxygenated monoterpenes, mixture design, molecular docking, virulence factors, bio-preservation, chemometric analysis

## Abstract

During the last few decades, there has existed an increased interest in and considerable consumer preference towards using natural and safe compounds derived from medicinal plants as alternatives to synthetic preservatives to combat microbial pathogenicity. In this regard, the present study investigated the possible synergistic interactions of the anti-foodborne bacterial capacity of linalool (L), eucalyptol (E), and camphor (C). The antibacterial synergistic effect was determined against *Staphylococcus aureus*, *Listeria monocytogenes*, *Salmonella enterica* Typhimurium, and *Escherichia coli*. The optimal predicted mixture showed the highest antibacterial activity at 33.5%, 33.2%, and 33.4% of L, E, and C, respectively. Molecular docking simulations displayed that the studied monoterpenes have effective antibacterial inhibitory effects by impeding specific virulence factors such as sortase A, listeriolysin O, L, D-Transpeptidase, and polyphosphate kinase. The selected triple combination of L, E, and C was applied as a natural preservative in minced chicken breast meat. In this regard, 1 MIC (16 µg/mL), 1.5 MIC (24 µg/mL), and 2 MIC (32 µg/mL) of *L. monocytogenes* were used, and the microbiological, physicochemical, and sensory analyses were monitored for 14 days of storage at 4 °C. The L/E/C mixture at different levels could delay lipid and protein oxidation, inhibit the microorganisms, and maintain the sensory attributes. Additionally, by using chemometric tools, strong connections between physicochemical properties, microbiological parameters, and organoleptic attributes were established. Concisely, this research confers the importance of the use of blended monoterpenes and highlights their antibacterial mode of action, effectiveness, and synergistic effects as a powerful and safe bio-preservative formulation in chicken meat products.

## 1. Introduction

Chicken meat is a popular and preferable essential part of human beings’ daily diet owing to its high digestibility, healthier nutritional properties, and desirable organoleptic attributes [1]. However, chicken-meat-based products are thoroughly susceptible to microbial contamination, spoilage, as well as lipid and protein oxidation reactions [2]. Thus, there is a growing trend and an excessive demand by consumers towards using natural plant-based preservatives owing to their safety, effectiveness, and ability to enhance the storage stability and physiological functionalities of chicken meat products as compared to synthetic additives, which display doubtful suitability along with toxicological and harmful effects [3,4].

Linalool (L), eucalyptol (E), and camphor (C) are natural compounds largely found and derived from a variety of plants such as those of the *Lauraceae* and *Lamiaceae* botanical families [5,6]. These valuable compounds are classified as generally recognized as safe by the Food and Drug Administration (FDA) and commonly utilized as value-added ingredients for nutraceutical, pharmaceutical, and cosmeceutical purposes owing to their various beneficial properties, including their antibacterial, antioxidant, antifungal, anti-inflammatory, and many other health-promoting activities [7]. However, it should be noted that the difference between safety and toxicity is tightly related to the administrated dose (i.e., it is dose-dependent). Wojtunik–Kulesza [8] indicated that the lethal dose of camphor ranged from 50 to 500 mg/kg in adults. Bhowal and Gopal [9] estimated that the lethal dose of eucalyptol could range from 0.05 to 0.5 mL/kg. An et al. [10] discussed the use of linalool as a therapeutic alternative with doses up to 100 mg/kg, proving its low toxicity. Previous research evidence has demonstrated that essential oils (EO) bearing L, E, and C could have various antibacterial mechanisms of action by inhibiting genetic material synthesis, cell wall efflux pumps, bacterial energy production, and metabolism and quorum sensing, as well as causing damage to the bacterial cell membrane [11]. Additionally, these oxygenated monoterpenes showed a great versatility of application in the food industry and related sectors and were applied as preservatives, stabilizers, or biocontrol agents with ecofriendly or green appeal to ensure the microbiological and oxidative stability of food products due to their effective biological characteristics as well as their pleasant odor and flavor [12]. They have also has been suggested as promising alternatives to be included in diet supplementation, as animal feed additives, bioactive food packing development, and bioinsecticides [13].

The employment of statistical methods such as simplex–centroid mixture design (SCMD) as an optimization model has allowed scientists to highlight the possible synergistic effects of compounds. It has also facilitated the prediction of pertinent models that could provide further explications by minimizing the number of experiments [14]. Several studies have focused on the enhancement of the antimicrobial activity of essential oil components via the development of combinatorial interactions [15]. These interactions may create synergistic effects that could improve their antimicrobial properties by decreasing the minimum inhibitory concentration, reducing their undesirable side effects and their negative organoleptic attributes on food [16].

On the other hand, targeting bacterial virulence factors has attracted pervasive attention as a promising approach to overcoming bacterial resistance [17]. Antivirulence agents have the potential to disarm pathogens and to attenuate virulence by begetting novel antibacterial mechanisms distinct to classical bacterial pathways [18]. In this context, computational techniques such as the molecular docking approach were invented to simulate the possible complexes and interactions between the bioactive compounds and bacterial-specific target enzymes that could be applied in the development of novel natural preservatives [19]. These predictions could provide credible results and could efficaciously save time and costs as compared to experimental assays. In the application of molecular docking in natural products, novel target simulations can facilitate the mission of justifying the traditional uses of natural components, as well as identifying new uses, alongside their introducing into different food matrices [20]. The usage of SCMD and molecular docking methods in the formulation of new biopreservative agents provides the advantages of formulating a powerful blend composed of L, E, and C with the best proportions. These methods also facilitate the investigation and identification of the exact bacterial targets and mechanisms of action in a short time, with lower cost and smaller error probability. Therefore, these mathematical algorithms could reduce animal harm, limit environmental pollution, and enhance the reproducibility, robustness, and versatility of experiments.

This research work aimed to evaluate the antibacterial activities of L, E, and C and to underline their synergistic effect using the SCMD method. This study focused on the prediction and optimization of their antibacterial potential against various foodborne and pathogenic bacteria in order to extend the shelf life of minced chicken breast meat. In addition, an in silico study was performed using a molecular docking approach to spotlight the possible mechanisms of action induced by the tested monoterpenes against several virulence factors, including sortase A of *S. aureus*, listeriolysin O of *L. monocytogenes*, L, D-Transpeptidase of *S. enterica* Typhimurium, and polyphosphate kinase of *E. coli*. On the other hand, the optimized mixture was applied to extend the shelf life of minced chicken breast meat samples. Microbiological, physicochemical, and sensory analyses were assessed during 14 days of storage at 4 °C. Finally, chemometric analysis, including principal component analysis (PCA), was performed to allow for the detection of relationships between the analyzed parameters over the storage days. The present study offers a special focus on the elucidation of the multitarget mechanisms of action of oxygenated monoterpenes that are still lacking in the literature and are still not completely developed. It shows their combinational effects and interactions when applied in food matrices, including chicken meat preservation.

## 2. Materials and Methods

### 2.1. Bioactive Compounds

Linalool (L) (C_10_H_18_O) (3,7-Dimethyl-1,6-octadien-3-ol), camphor (C) (C_10_H_16_O) (1,7,7-Trimethylbicyclo[2.2.1]heptan-2-one), and eucalyptol (E) (C_10_H_18_O) (1,3,3-Trimethyl-2-oxabicyclo[2.2.2]octane) were purchased from Sigma-Aldrich (St. Louis, MI, USA). The purity of these compounds was 97%, 98%, and 99%, respectively.

### 2.2. Antibacterial Assays

#### 2.2.1. Bacterial Strains

Two Gram-negative bacteria—*E. coli* ATCC 8739 and *S. enterica* Typhimurium ATCC 14028—and two Gram-positive bacteria—*S. aureus* ATCC 6538 and *L. monocytogenes* ATCC 19117—from the collection of the Laboratory of Microbial Biotechnology, Enzymes, and Biomolecules (LMBEB), Center of Biotechnology of Sfax (CBS), Tunisia, were used to investigate the antibacterial potential of the selected oxygenated monoterpenes. Bacterial cultures were prepared in a Luria–Bertani (LB) agar medium and incubated overnight at 37 °C according to the method reported by Elhadef et al. [21].

#### 2.2.2. Determination of Minimum Inhibitory Concentration (MIC)

The minimum inhibitory concentration (MIC) is defined as the lowest concentration of an antibacterial agent that could impede the growth of a microorganism [22]. An MIC assay test was carried out using sterile 96/U-PP Eppendorf microplate (Greiner, Nürtingen, Germany). The dimethyl sulfoxide (DMSO) at 1% was used for the dissolution of the pure compounds (linalool, eucalyptol, and camphor). It also served as a negative control as no detrimental effect on bacterial growth was observed at this concentration (<5%) [23].

For each test, the L, E, and C compounds were deposited into the respective well to perform a two-fold serial dilution from the original sample with a final volume of 100 μL per well. Then, 10 μL of cell suspension of the studied microorganism was added to a final inoculum concentration of 10^6^ colony-forming unit (CFU)/mL and incubated overnight at 37 °C. A volume of 25 µL of thiazolyl blue tetrazolium bromide (MTT) at 0.5 mg/mL was added to the wells and incubated at room temperature for 30 min [24].

### 2.3. Mixture Design

The SCMD model was chosen to optimize and determine the synergistic antibacterial effect of the three oxygenated monoterpenes. The factors presented the proportion of each compound in the mixture. Ten experiments were determined with various L, E, and C mixtures. The obtained experiments were distributed as follows: the three compounds in the triangle’s vertices included the Exp 1, 2, and 3; the binary mixtures 0.5/0.5 included the Exp 4, 5, and 6; the compounds in equal proportion were considered as the Exp 7; and the ternary combinations included the Exp 8, 9, and 10, as mentioned in Supplementary Table S1.

Y = ∑βi × A+∑βij × A × B + ∑βijk × A × B × C + ϵ(1)

Linear, quadratic, and special cubic regression models were employed to assess the variations of all the effects and their interactions in each response (Equation (1)).

Y is the predicted response, while βi, βij, and βijk correspond to the regression coefficients for linear, binary, and ternary interaction effect terms, respectively. A, B, and C are the variables, and ε is the random error. The software Minitab 16 was used for experimental design and data analysis.

The optimization via the SCMD model facilitated the obtention of a powerful formulation among the tested mixtures, giving the highest antibacterial activity, which is marked as the minimum inhibitory concentration of the mixtures (MICm). The MICm of the L, E, and C combinations were carried out according to the same protocol previously described in Section 2.2.2.

### 2.4. In Silico Study of the Antibacterial Properties of the Studied Bioactive Compounds

#### 2.4.1. Ligands Preparation

The simplified molecular input line entry systems (SMILES) strings of L, E, and C were downloaded from the PubChem database [25] and converted into 3D structures using Corina Demo webserver [26].

#### 2.4.2. Bacterial Targets Selection and Binding Site Prediction

In silico antibacterial activities of L, E, and C compounds were evaluated against four antivirulence targets, namely, *S. aureus* NCTC 8325 and *L. monocytogenes* strain 10403S, *S. enterica* Typhimurium ATCC 700720, and *E. coli* K12. All protein receptors’ FASTA sequences, including sortase A (PDB: 1T2P), Listeriolysin O (PBD: 4CDB), L, D-Transpeptidase (PBD: Q87QB5), and polyphosphate kinase (PDB: 1XDO), respectively, were obtained from Uniprot database [27]. Only L, D-Transpeptidase of *S. enterica* Typhimurium was subjected to the Swiss model [28] for the molecular homology approach as no crystallographic structure was available. Binding site residues of the selected receptors were predicted using the “reverse template comparison vs. Structure in PDB approach” [29] and proceeded to molecular docking to identify and represent how the tested monoterpenes can be used to tackle microbial resistance and prevent food contamination.

#### 2.4.3. Molecular Docking Simulations and Interaction Profiles Visualization

In this section, molecular docking simulations were carried out against the selected virulence factors using the Autodock Vina software version 1.2.0 [30]. All steps related to molecular docking simulations were performed according to the method previously described by Akermi et al. [31]. All water molecules were removed, then Kollman and Gasteiger charges and polar hydrogen atoms were assigned to the selected protein receptors and saved in a pdbqt file format. A grid box was built around the binding sites of the receptor proteins by making a grid box of 126 × 126 × 126 in size with a grid spacing of 0.375 Å with different coordinates of sortase A of *S. aureus* (X = 0.381; Y = 4.585; and Z = 12.069), Listeriolysin O of *L. monocytogenes* (X = −2.978; Y = 16.77; and Z = −46.153), YcbB of *S. enterica* Typhimurium (X = 24.481; Y = −39.943; and Z = 12.254), and PPK of *E. coli* (X = −40.333; Y = 85.762; and Z = 41.615). The most stable conformations of the ligands with the highest affinities were selected and ranked based on the lowest binding energies. Finally, the interaction profiles were visualized and analyzed using BIOVIA^®^ Discovery Studio^®^ version 16.1.0 (Dassault Systems BIOVIA, 2016, San Diego, CA, USA).

### 2.5. Analysis of Raw Chicken Breast Meat Samples

#### 2.5.1. Samples Preparation

The fresh raw chicken breast meat was purchased from a local market located in the region of Sfax, Tunisia. The chicken meat was minced using a sterile meat grinder with a 3 mm-diameter mincing plate. The obtained quantity was equally divided into five lots (2250 g for each sampling day (0, 3rd, 7th, 10th, and 14th)). The obtained samples were kept in sterile plastic bags and stored under aseptic conditions at 4 °C.

The first sample was considered as a control (untreated sample), the second one was employed as a positive control and treated with 0.01% of butylated hydroxytoluene (BHT) (a synthetic preservative used in food industry) [32]. BHT was classed among the antioxidants explicitly approved for use in foods by the U.S. Food and Drug Administration (FDA) and the U.S. Department of Agriculture (USDA) [33,34,35]. This synthetic antioxidant has strict limits on its usage levels and is specific to different products. The limit of BHT in raw beef meat was 0.01% individually [36]. On the other hand, BHT was added to meatball formulation at 0.01% concentration, which was allowed by the Turkish Food Codex (Regulation on Food Additives) [37].

On the other side, the optimized formulation was employed in the proportions of 33.5% for L, 33.2% for E, and 33.4% for C. The treatment of the three other samples corresponds to (1-L/E/C), (1.5-L/E/C), and (2-L/E/C), which were, respectively, 16 µg/mL (containing 5.36 µg of L, 5.312 µg of E, and 5.344 µg of C), 24 µg/mL (containing 8.04 µg of L, 7.968 µg of E, and 8.016 µg of C), and 32 µg/mL (containing 10.72 µg of L, 10.624 µg of E, and 10.688 µg of C). The application of L/E/C mixture at different concentrations was undertaken via their direct addition to the samples and subsequent homogenization under the microbiological hood to avoid any external contamination. All the samples were then aseptically stored during 14 days at 4 °C. A total of 75 trials (5 × 3 × 5) were employed as follows: five treatments (control, BHT, (1-L/E/C), (1.5-L/E/C), and (2-L/E/C)) for three sub-samples over the sampling period (five storage days: 0, 3, 7, 10, and 14 days).

#### 2.5.2. Microbiological Analyses

According to Smaoui et al.’s [38] protocol, microbiological analyses consisted of the evaluation of aerobic plate counts (APC) by incubating plate count agar (PCA) at 30 °C for 48 h [39]. Additionally, psychrotrophic total counts (PTC) were assessed by incubating PCA medium at 7 °C for 10 days [40], and Enterobacteriaceae counts (EC) were incubated at 37 °C for 24 h using a violet red bile glucose agar (VRBG) medium [41]. All the microbiological data were converted into logarithms of the number of colony-forming units (CFU/g).

#### 2.5.3. Physiochemical Analyses

##### pH Analysis

A total of 5 g of minced chicken breast meat was taken from each sample and homogenized in 50 mL of distilled water (pH 7.00) then filtered and measured using a pH meter (pH210 Microprocessor pH Meter, HANNA instruments, Kehl am Rhein, Germany) at each sampling day [42].

##### Evaluation of Protein/Lipid Oxidation

Concerning lipid oxidation, peroxide values (PV) of the chicken breast meat samples were assessed using the Folch method and the obtained results were expressed in milliequivalents of peroxide per kg of meat [43]. On the other side, the conjugated dienes (CDs) values were measured according to the protocol of Cagdas and Kumcuoglu [44]. In this sense, 1 g was taken from each sample and mixed with 10 mL of distilled water. Subsequently, 5 mL of a 3:1 (*v*/*v*) ratio of hexane and isopropanol solvent was used to dissolve 0.5 mL of the mixture previously prepared. The supernatants’ absorbance was measured at 233 nm after 5 min of centrifugation at 2000× *g*. CD values were reported in µmol/mg of meat sample. The thiobarbituric acid reactive substances (TBARS) values were determined by combining 2 g of meat with 100 µL of butylated hydroxytoluene (1 g/L) and 16 mL of trichloroacetic acid (TBA) (50 g/L). The obtained mixture was then filtered, and 2 mL of filtrate and 2 mL of TBA solution (2 mol/L) were combined. The mixture was heated at 70 °C for 15 min before cooling, and the absorbance was read at 532 nm. TBARS values were expressed as milligrams of malondialdehyde (MDA equivalent) per kilogram sample following the method reported by Mtibaa et al. [45].

Finally, the protein oxidation process was evaluated by measuring protein carbonyl content (CC) in nmol carbonyl/mg of protein according to the method detailed by Mtibaa et al. [45].

##### Assessment of Sensory Attributes

With a view to investigate the sensory characteristics of raw minced chicken breast meat samples during 0, 3, 7, 10, and 14 days of refrigerated storage, 30 laboratory panelists (15 females and 15 males) were chosen among graduate students and administrative staff of the center of Biotechnology of Sfax using the following criteria: ages between 20 and 48, non-smokers, and those who consume beef meat products regularly. Each panelist performs three different assays for treated and untreated samples to periodically check and evaluate the odor, color, appearance, and overall acceptability (OA) of all the samples. The different samples were individually presented, at room temperature (25 °C), in small covered porcelain dishes in a separate area. The experimental approach and the samples were blind-coded with 3-digit random numbers and presented in individual booths to each panelist for evaluation. Therefore, a hedonic scale of 9 points (9 = like extremely; 8 = like very much; 7 = like moderately; 6 = like slightly; 5 = neither like nor dislike; 4 = dislike slightly; 3 = dislike moderately; 2 = dislike very much; 1 = dislike extremely) was used [46].

### 2.6. Statistical Analyses

Experimental data were established using SPSS 19, (SPSS UK Ltd., Woking, UK). The variables were subjected to one-way analysis of variance (ANOVA), and the level of significance was analyzed using the Tukey test at (*p* < 0.05). Subsequently, principal component analysis (PCA) was performed using XLSTAT software for Windows (v.2014.1.08, Addinsoft, New York, NY, USA). All tests were conducted in triplicate.

## 3. Results and Discussion

### 3.1. Mixture Design

In this study, the main reason justifying the selection of linalool, eucalyptol, and camphor was the fact that the combination of oxygenated monoterpenes could increase their ratio and, accordingly, their effectiveness in producing a synergistic effect against pathogenic and foodborne bacteria [47]. Therefore, the SCMD model was designed to find a powerful formulation that could inhibit bacterial growth and proliferation.

Experimental and predicted responses of antibacterial activities (MICs of *S. aureus*, *S. enterica* Typhimurium, *E. coli*, and *L. monocytogenes*) were elucidated (Supplementary Table S1). Findings showed the variation of bacteria’s susceptibility to various combinations. The lowest MIC was 10.5 µg/mL against both *S. enterica* Typhimurium and *L. monocytogenes*, while *S. aureus* and *E. coli* exhibited the lowest MICs values, equal to 21 and 42 µg/mL, respectively.

**Figure 1:**
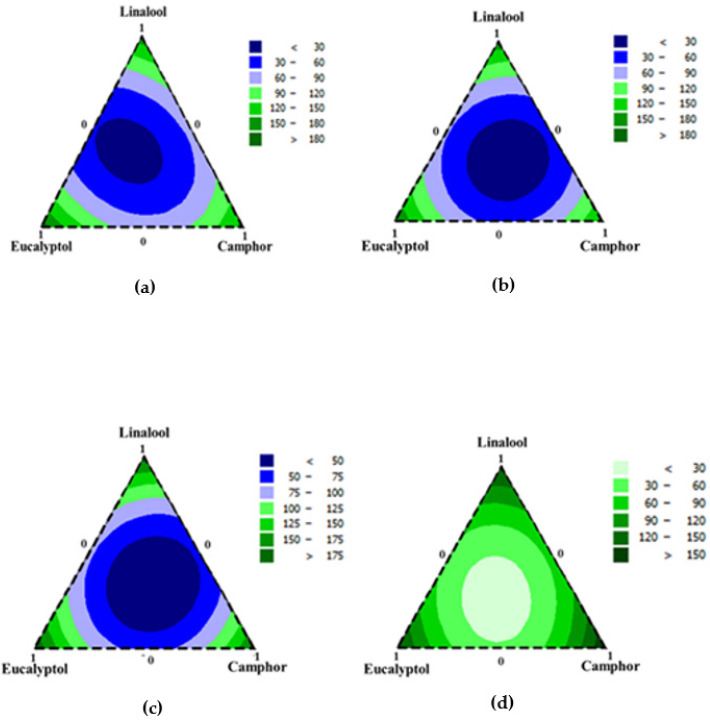
Contour plots for the effects of different compounds mixtures on *S. aureus* (**a**), *S. enterica* Typhimurium (**b**), *E. coli* (**c**), and *L. monocytogenes* (**d**). Results are expressed by MIC (µg/mL).

The results of responses regression models (Supplementary Table S2) and the corresponding contour plots are traced in Figure 1a–d. These contour plots consist of three vertices reflecting the individual components, and the triangle edges represent the components in their binary mixture. The dark blue regions in all response contour plots indicate the minimum concentration. The effectiveness and the relevance of the models in predicting the responses were evaluated by R2. MICs of *S. aureus*, *S. enterica*, *E. coli*, and *L. monocytogenes* depicted R^2^ values of 92.22, 95.46, 95.94, and 91.72%, respectively.

#### 3.1.1. Effect of the Mixture on the Antibacterial Activities

- Anti-*S. aureus* Activity

The MIC of *S. aureus* ranged between 21 and 168 µg/mL. In fact, the *p*-value of the quadratic interaction (A × B) was significant, with *p* = 0.029 (Supplementary Table S2). This finding was in good agreement with Karaca et al. [48], who reported that major components of *Lavandula latifolia* essential oil were analytically determined as L (45.2%) and E (25.6%). These authors reported an antibacterial activity against *S. aureus* (MIC = 2.5 mg/mL). Another study showed that *Salvia fruticose* essential oil containing eucalyptol (29.04%) and camphor (21.32%) as major compounds has an efficient antibacterial activity against *S. aureus*, *B. subtilis*, and *E. coli*, and stipulated MIC values ranging from 56.2 to 146 μg/mL for all pathogens [49]. The mathematical regression model for expressing the response is mentioned as follows:

MIC of *S. aureus* (µg/mL) = 168.4 × A + 195.9 × B + 161 × C − 591.5 × AB − 373.3 × AC − 402.2 × BC − 92.1 × ABC. (2)

- Anti-*S. enterica* Typhimurium Activity

The MIC of *S. enterica* Typhimurium varied between 10.5 and 168 µg/mL (Supplementary Table S1). Results showed that the binary mixture of L (50%) and E (50%) exhibited the highest anti-*S. enterica* Typhimurium activity (10.5 µg/mL). The quadratic interactions were significant (*p* < 0.05), with *p*-values of 0.018, 0.014, and 0.016 for A × B, A × C, and B × C, respectively (Supplementary Table S2). Tardugno et al. [50] reported that *Lavandula x intermedia* EO exerted an effective anti-*Salmonella* sp. activity, with MIC = 10 mg/mL. This fact could be explained by the presence of high amounts of L (61.98%) and C (10.30%). In addition, Rodrigues et al. [51] suggested that the high level of antibacterial activity of *Thymus mastichina* (EO), especially against *Salmonella* sp., was attributed to the high percentage of camphor. The mathematical regression model is represented as follows:

MIC of *S. enterica* (µg/mL) = 173.4 × A + 187.6 × B + 162.2 × C − 558.1 × AB − 608.8 × AC − 580.5 × BC + 1706.8 × ABC. (3)

- Anti-*E. coli* Activity

Results for anti-*E. coli* activity indicated that the binary and ternary mixture yielded the lowest MIC, equal to 42 µg/mL (Supplementary Table S1). The quadratic interactions were significant (*p* < 0.05), with *p*-values equal to 0.022, 0.015, and 0.017 for A × B, A × C, and B × C, respectively (Supplementary Table S2). Similar behavior of the antimicrobial activity of cinnamon oil was attributed to L and C components [52]. Focusing on linalool, it was determined that its addition to some EOs (*Thymus vulgaris* and *Syzygium aromaticum*) enhanced the spectrum of their antimicrobial activity [53]. A study of Puvača et al. [54] reported that essential oils rich in linalool, including those of the tea tree plant, could have an antibacterial activity against *E. coli*, with a recorded MIC equal to 3.1 mg/mL. The generalized polynomial equation considered is represented as follows:

MIC of *E. coli* (µg/mL) = 186.6 × A + 197.8 × B + 181.4 × C − 471.2 × AB − 539.9 × AC − 517.5 × BC + 507.2 × ABC. (4)

- Anti-L. monocytogenes Activity

Findings for the MIC of *L. monocytogenes* ranged between 10.5 and 168 µg/mL. The lowest MIC concentration was observed at 33.3% of each compound. The *p*-value of the quadratic interaction (B × C) was significant, with *p* = 0.049 (Supplementary Table S2). Our study showed better results than those obtained by Nafis et al. [55], who reported that *Lavandula angustifolia* EO containing 39.05% of E and 24.21% of C displayed antibacterial activity against *S. aureus* (15.96 mg/mL), *E. coli* (7.93 mg/mL), and *Salmonella* sp. (7.93 mg/mL). The previously cited research confirmed that the presence of oxygenated monoterpenes derived from essential oils could have an efficient impact and synergetic effect on controlling pathogenic bacteria by inhibiting their growth and proliferation. The mathematical regression model is mentioned as follows:

MIC of *L. monocytogenes* (µg/mL) = 155.7 × A + 159.5 × B + 176.1 × C − 361.6 × AB − 328.3 × AC − 484.9 × B × C − 926.5 × A × B × C. (5)

Indeed, Figure 1a–d indicated that the ternary mixture L/E/C allowed for high antibacterial activities.

#### 3.1.2. Mixture Design Optimization

**Figure 2:**
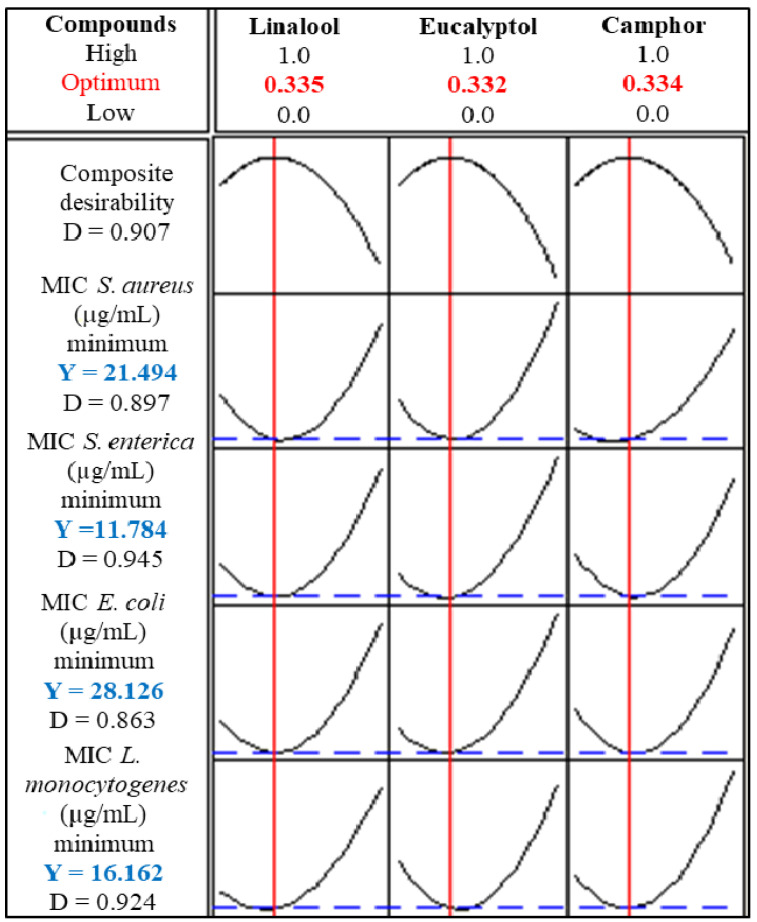
Response optimizer at the optimum conditions for the maximum response.

A numerical optimization method of the various mixtures was employed to identify the maximum number of responses. To maximize each reaction, the proportions of compounds were raised to their ideal levels. This approach searches simultaneously for a collection of factor values that satisfy the requirements of the design. In fact, this investigation indicated that the ternary combination with percentages of 33.5, 33.2, and 33.4% of L/E/C led to maximum anti-*S. aureus* (21.49 µg/mL), anti-*S. enterica* Typhimurium (11.78 µg/mL), anti-*E. coli* (28.12 µg/mL), and anti-*L. monocytogenes* (16.16 µg/mL) activities (Figure 2).

### 3.2. In Silico Evaluation of the Antibacterial Potential of L, E, and C

It is well known that deterioration and foodborne pathogens, including *S. aureus*, *S. enterica*, *L. monocytogenes*, and *E. coli*, could deteriorate food quality by releasing extracellular enzymes, leading to a myriad of diseases [56]. For that reason, it is crucial to find possible novel ways to inhibit their proliferation and to overcome their virulence. In this regard, we focused on the investigation of the antibacterial potential of linalool, eucalyptol, and camphor against selected virulence factors involved in the bacterial colonization and invasion of, as well as adhesion to the host, alongside biofilm and toxin formation. The molecular homology results of the stereochemical quality parameters related to the YcbB virulence protein factor of *S. enterica* Typhimurium showed an acceptable identity percentage > 30% (84.44%), a pertinent Ramachandran plot favoring regions > 90% (93.3%), and a satisfactory QMEAN value (−1.36). Accordingly, a virtual screening approach of the selected bioactive compounds was applied against staphylococcal Sortase A protein (SrtA), L, D-Transpeptidase (YcbB) of *S. enterica* Typhimurium, pore-forming Listeriolysin O (LLO) of *L. monocytogenes*, and the polyphosphate kinase (PPK) of *E. coli* using molecular docking approach (Table 1).

**Figure 3:**
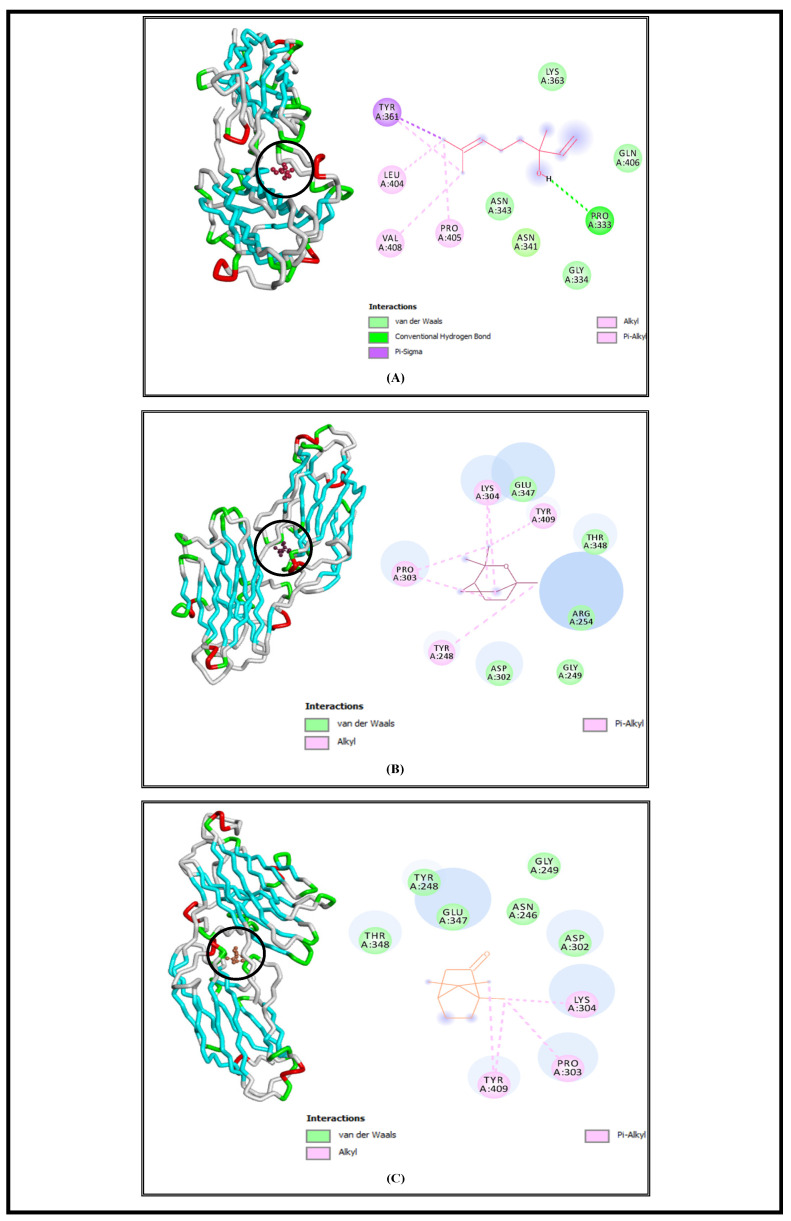
Interaction complexes of linalool (**A**), eucalyptol (**B**), and camphor (**C**) with the Sortase A protein of *S. aureus*.

**Figure 4:**
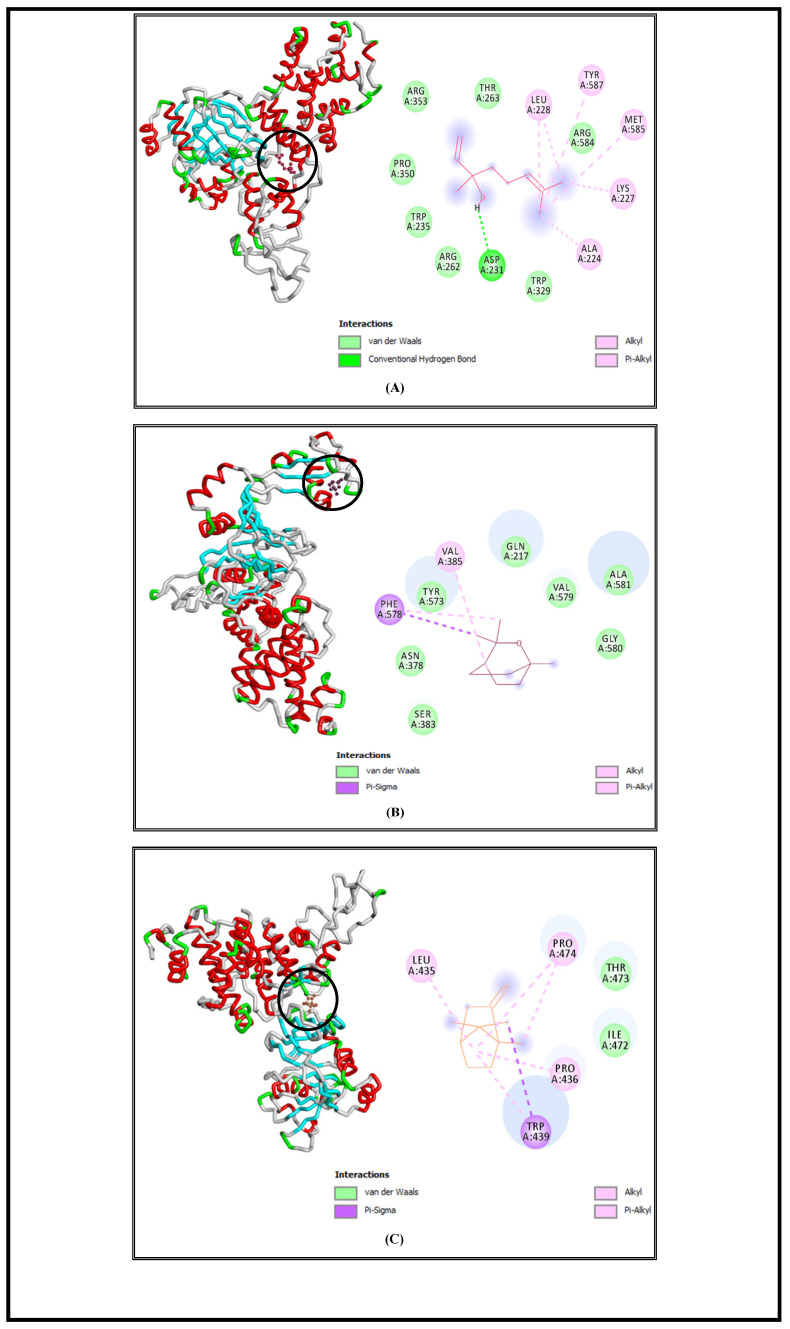
Interaction complexes of linalool (**A**), eucalyptol (**B**), and camphor (**C**) with the YcbB protein of *S. enterica* Typhimurium.

**Figure 5:**
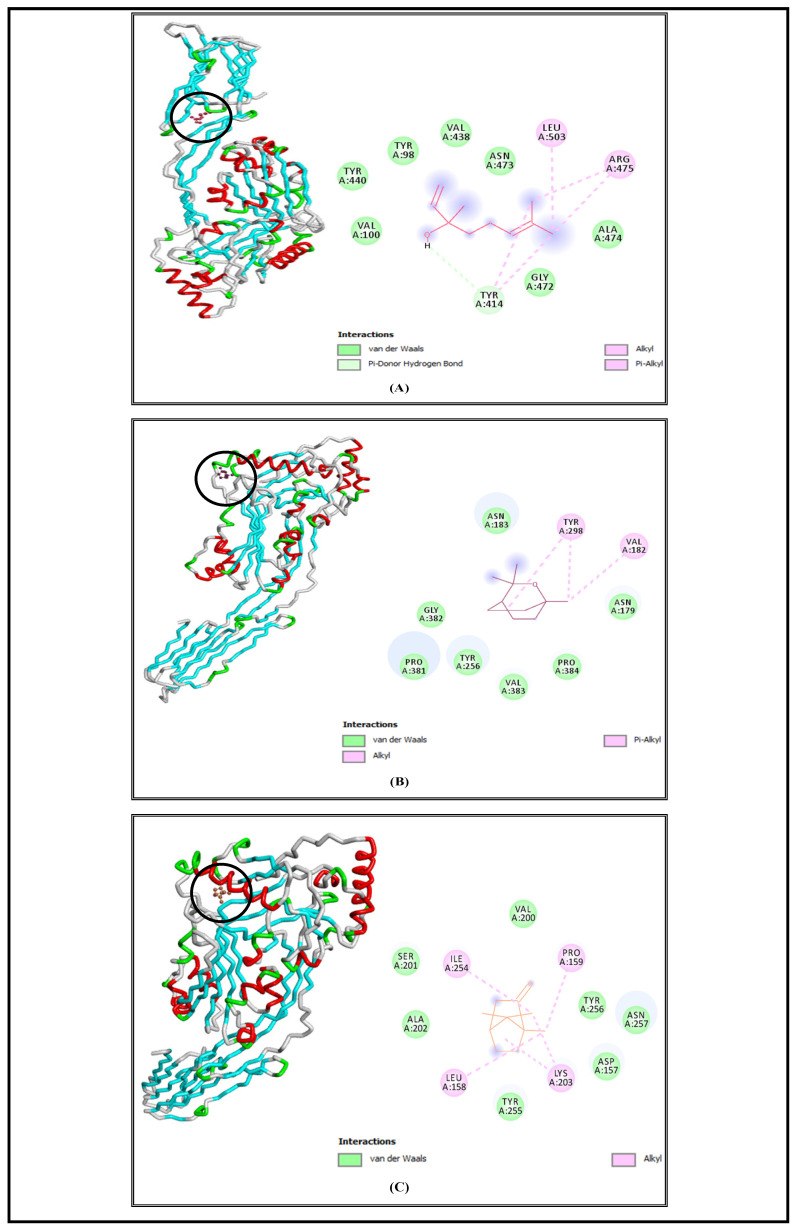
Interaction complexes of linalool (**A**), eucalyptol (**B**), and camphor (**C**) with the listeriolysin O protein of *L. monocytogenes*.

**Figure 6:**
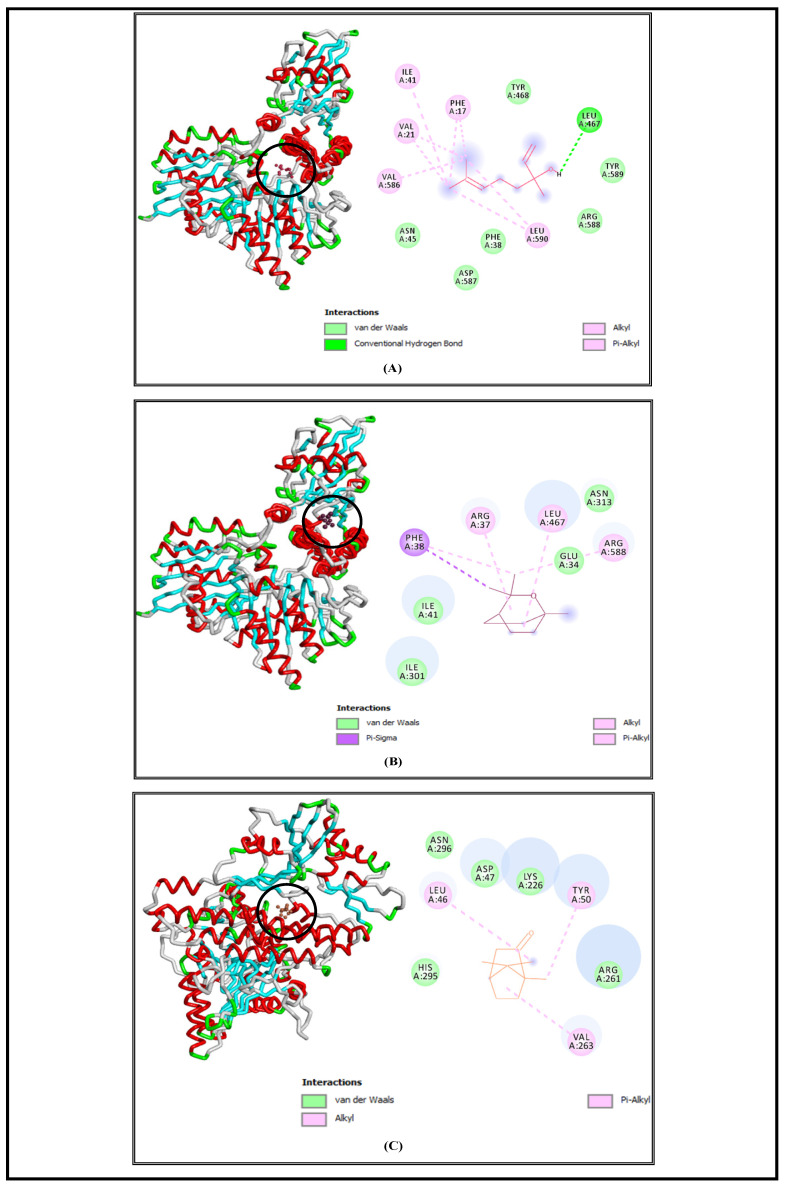
Interaction complexes of linalool (**A**), eucalyptol (**B**), and camphor (**C**) with the PPK protein of *E. coli*.

SrtA is a bacterial transpeptidase protein with a critical role in the pathogenesis of *S. aureus* and which is convoluted in the adhesion and invasion of host cells as well as biofilm formation and signaling [57]. The molecular docking simulations demonstrated that L, E, and C had high affinities to SrtA and showed low free energies of binding at −6.5 Kcal/mol, −6.3 Kcal/mol, and −6.3 Kcal/mol, respectively (Table 1). Based on the obtained results, we suggested that the studied bioactive compounds could inhibit the activity of the SrtA enzyme by binding to its active site. Similar findings were demonstrated by another in silico study, which displayed that hibifolin attenuated the pathogenicity of *S. aureus* by inhibiting SrtA activity at free energy of binding equal to −6.8 Kcal/mol [58]. The interaction profiles representing the binding mode of the tested molecules against the selected bacterial virulence factors active sites are elucidated in Figure 3, Figure 4, Figure 5 and Figure 6, and all the interaction details are summarized in Table 1.

YcbB of *S. enterica* Typhimurium is a key virulence factor involved in several pathogenicity mechanisms, including β-lactam resistance, bacterial outer membrane defect rescue, and typhoid toxin release. The calculation results revealed that L, E, and C displayed a potential antivirulence activity against *S. enterica* Typhimurium by inhibiting (YcbB) activity. These compounds showed free energies of binding equal to −6.2 Kcal/mol, −5.9 Kcal/mol, and −5.9 Kcal/mol, respectively (Table 1). A previous study indicated that cyclovalone showed potent antibacterial activity (−7.6 Kcal/mol) against the selected target (YcbB) as compared to FDA-approved antibiotics carbapenem (−4.3 Kcal/mol) and cephalosporin (−6.8 Kcal/mol) [59].

On the other hand, LLO is an imperative virulence factor produced by *L. monocytogenes*. It was reported that LLO supports bacterial pathogenicity by mediating its escape from the host’s immune system [60]. Molecular docking results of listeriolysin O complex with L, E, and C indicated that all three molecules have a potent antivirulence activity against the studied target. Their free energies of binding were approximately −6.1 Kcal/mol, −5.6 Kcal/mol, and −5.6 Kcal/mol, respectively (Table 1). An earlier study demonstrated that Lutein (−6.0 Kcal/mol) and Fisetin (−4.7 Kcal/mol) could block the oligomerization of LLO [61].

PPK is a pivotal enzyme involved in motility, quorum sensing, and biofilm formation of pathogenic *E. coli* [62]. Molecular docking results showed that the studied bioactive molecules could inhibit *E. coli* (PPK) activity. Their (ΔG) were estimated to be −6.7 Kcal/mol, −6.6 Kcal/mol, and −6.5 Kcal/mol, respectively (Table 1). Another study indicated that eucalyptol binds efficiently with the active site of the *E. coli* protein, and it showed a higher activity than gentamicin, with a binding energy of −5.72 kcal/mol and −5.55 kcal/mol [63].

In Table 1, the interaction profile details of all the tested molecules against the selected bacterial virulence factors are elucidated.

Linalool complex with the (SrtA) protein receptor of *S. aureus* displayed the presence of alkyl and Pi–alkyl interactions via LEU404, VAL408, and PRO405; van der Waals interactions with LYS363, GLN406, GLY334, ASN341, and ASN343; one conventional hydrogen bond with PRO333; and Pi–sigma interaction with TYR361 (Figure 3A). Additionally, eucalyptol showed various interactions with the selected virulence factor of *S. aureus.* It indicated the existence of alkyl and Pi–alkyl interactions with TYR248, PRO303, LYS304, and TYR409, and van der Waals interactions via GLU347, THR348, ARG254, GLY249, and ASP302 (Figure 3B). The complex of camphor with sortase A receptor was elaborated via alkyl and Pi–alkyl interactions with LYS304, PRO303, and TYR409, and van der Waals interactions with THR348, TYR248, GLU347, ASN246, GLY249, and ASP302 (Figure 3C).

Linalool complexed with L, D-Transpeptidase (YcbB) of *S. enterica* Typhimurium showed alkyl and Pi–alkyl interactions with LEU228, TYR587, MET585, LYS227, and ALA224; van der Waals interactions with ARG584, THR263, ARG353, PRO350, TRP235, ARG262, and TRP 329; and one conventional hydrogen bond with ASP231 (Figure 4A).

The complex of eucalyptol and the L, D-Transpeptidase (YcbB) of *S. enterica* Typhimurium revealed the existence of alkyl and Pi–alkyl interactions with VAL385; van der Waals interactions with GLN217, VAL579, ALA581, GLY580, SER383, ASN378, and TYR573; and a Pi–sigma interaction via PHE 587 (Figure 4B).

The virulence factor receptor of *S. enterica* Typhimurium complexed with camphor indicated the existence of alkyl and Pi–alkyl interactions with the residues PRO474, PRO436, and LEU435; van der Waals interactions with THR473 and ILE472; and a Pi–sigma interaction via TRP439 (Figure 4C).

Therefore, the three tested compounds were found to have an effective multitarget antibacterial activities against pathogenic bacteria by inhibiting specific virulence factors such as sortase A of *S. aureus*, listeriolysin O of *L. monocytogenes*, L, D-Transpeptidase of *S. enterica* Typhimurium, and polyphosphate kinase of *E. coli*. However, further experiments are still needed to support the in silico design and to better establish the antibacterial mechanism of action. In this regard, we will seek in subsequent studies to perform multiple in vitro assays.

### 3.3. Application of Triple Combination of L, E, and C on Raw Minced Chicken Breast Shelf life

The optimized mixture (33.5% L, 33.2% E, and 33.4% C) at different MICs values (1, 1.5, and 2 MICs of *L. monocytogenes*) was used to assess its effectiveness in the extension of the shelf life of minced chicken breast meat samples. The choice of the concentrations (1, 1.5, and 2 MIC) was employed to define the exact and the efficient concentration to preserve the studied chicken meat product. Some studies investigated the 1 MIC, 2 MIC, 3 MIC, and/or 4 MIC against *L. monocytogenes* to study their preservative potential on meat products. For instance, the impact of *Lobularia maritiman* essential oil was examined by Ben Akacha et al. [64]. These authors reported that the MIC value against *L. monocytogenes* was 19 µg/mL, and three concentrations—viz. 0.019 (1 MIC), 0.038 (2 MIC), and 0.076% (3 MIC)—were studied to explore their impact on ground meat beef preservation at 4 °C. Similarly, Smaoui et al. [65] examined three levels of *Mentha piperita* essential oils and investigated their impact on meat beef meat during 21 days at 4 °C. These latter correspond to 0.025 (1 MIC), 0.05 (2MIC), and 0.1 mg/mL (4 MIC) against *L. monocytogenes*.

It should be noted that *L. monocytogenes* was chosen based on several reasons related to its fast ability to contaminate various food matrices, especially meat products. Compared to other pathogens, *L. monocytogenes* possesses great heat resistance (thermotolerance), as well as a high capacity to grow at low temperatures [66]. Additionally, this bacterium can cause an invasive infection with a high mortality rate known as listeriosis after consuming contaminated chicken meat.

#### 3.3.1. Microbiological Analysis

The microbial load of APC, PTC, and EC of raw minced chicken breast meat were assessed during 14 days of storage at 4 °C (Table 2). Over the storage period, APC and PTC of the control samples significantly (*p* < 0.05) increased from 3.96 and 3.46 log CFU/g and reached 7.67 and 7.51 log CFU/g, respectively. Similarly, it was observed that EC attained 3.20 log CFU/g at the end of storage. Therefore, the untreated samples overpassed the limits of the shelf life (6.7 log CFU/g for APC and PTC and 2 log CFU/g for EC) declared by the Association of French Normalization Organization Regulation (AFNOR), indicating that the chicken meat became unfit for consumption [67]. However, APC, PTC, and EC growth rates significantly (*p* < 0.05) decreased in treated samples owing to the addition of the L/E/C mixture at different concentrations (16, 24, and 32 µg/mL). Interestingly, 2-L/E/C (at 32 µg/mL) displayed better antibacterial activity than the BHT. At the end of the storage days, the calculated bacterial growth rate related to the APC of all the samples revealed that the untreated sample showed the highest rate (48.37%) as compared to the positive-control BHT (30.28%) and the treated samples 1 L/E/C (35.29%), 1.5 L/E/C (32.99%), and 2 L/E/C (29.53%), which exhibited the lowest bacterial contamination rate among all the samples. Furthermore, PTC growth rate was estimated to be approximately 53.92% for the control sample, 38.32% for BHT, and the lowest for the treated samples (1 L/E/C (41.94%), 1.5 L/E/C (40.13%), and 2 L/E/C (36.97%)). Similar results were found by Peighambardoust et al. [68], who suggested that Yarrow plant EO, rich in eucalyptol and camphor, could block the growth of PTC (3.97–4.65 log CFU/g) in chicken meat until 15 days of storage. Previous studies suggested that these bioactive compounds could exert their bactericidal activities by interacting with lipid bilayers, resulting in multiple mechanisms of action, including the perturbation of cell membrane functionality and integrity, as well as the inhibition of bacterial enzymes involved in ATP production and genetic material replication and transcription [69,70]. Merghni et al. [71] demonstrated that eucalyptol could damage the membrane integrity and induce ROS-mediated oxidative stress in MRSA cells. Another study stated that linalool showed a strong inhibitory activity against *L. monocytogenes* by engendering cell damage, disturbing the central carbon metabolism, and affecting the bacterial respiratory metabolism [72]. On the other side, *Curcuma heyneana* essential oil containing eucalyptol and camphor as major compounds exhibited a potent antibacterial activity against *E. coli* by reducing biofilm formation and disrupting membrane integrity [73].

Hence, these findings could be explained by the fact that oxygenated monoterpenes exhibit an effective antibacterial inhibitory effect due to their low water solubility and the existence of an aromatic nucleus. Additionally, the obtained results have great interest in the field of food safety and preservation because the employment of synthetic preservatives can cause consumer health concerns. Accordingly, the use of natural biomolecules that could be safely applied as efficient preservative agents in the meat industry could be considered as a valuable approach. These natural additives constitute a serious challenge for controlling food borne pathogens by disarming microbial virulence factors, reducing the development of resistance, and limiting the death rate and toxi-infection diseases.

#### 3.3.2. Physiochemical Analysis

##### pH Values

The initial pH of all the samples was 5.79 (Table 3). It has been observed that no significant variation (*p* > 0.05) was detected between all pH values before the 7th day. However, the highest pH values were noticed to belong to the untreated sample, which significantly increased (*p* < 0.05) from 5.79 to 6.84 until the 14th day of storage. This pH rise could be explained by the formation and accumulation of alkaline compounds such as ammonia and volatile amines arising as metabolites from bacterial activities and growth [74]. Oppositely, no significant variation (*p* > 0.05) was detected until the 14th day, especially in chicken samples treated with 1.5-L/E/C and 2-L/E/C, and both showed better results than those treated with the synthetic preservative (BHT). The increase in the pH of untreated samples might be due to higher growth of microorganisms, the production of bacterial metabolites, and especially the deamination of proteins. However, the treated samples showed stable and acceptable pH values until the end of the storage period. These results could be attributed to the powerful antioxidant and antibacterial properties of the L/E/C mixture, which delayed the protein and lipid oxidation as well as the microbial proliferation. Consequently, the stabilization of the pH of the minced chicken breast meat samples was achieved by retarding the generation of volatile amino acids.

##### Evaluation of Lipid/Protein Oxidation

The lipid peroxidation process encompasses the steps of degradation of polyunsaturated fatty acids and the production of secondary metabolites, leading to undesirable effects such as the appearance of rancidity, the modification of texture and color, as well as the loss of the quality and the nutritional value of meat [75]. As described by Hadidi et al. [76], Lipid oxidation has three main stages, including the initiation that induces the formation of alkyl radicals as primary products. The second stage is the propagation of the formed alkyl with unsaturated fatty acids to generate hydroperoxides, which, in turn, form alkoxy radicals and hydroxyl radicals. The decomposition of these latter substances leads to the formation of aldehydes, ketones, and alkanes. Finally, completion is the last step, giving rise to non-radical compounds that could have detrimental effects on human health. The PV level of the control sample was 0.04 meq peroxides/kg and significantly increased (*p* < 0.05) until reaching 0.85 meq peroxides/kg at the end of storage in refrigerated condition (Table 3). However, it was significantly lower (*p* < 0.05) in treated samples than in the control sample (0.6–0.71 meq peroxides/kg), and the 2-L/E/C sample had the lowest PV content (0.43 meq peroxides/kg). Additionally, a progressive increase was noticed in CD values in the control sample (*p* < 0.05), while in the treated samples, a slight increase was detected. At the end of storage time, CD content was significantly (*p* < 0.05) the lowest in the 2-L/E/C sample (Table 3). On the other hand, the TBARS values of untreated samples were continuously and significantly (*p* < 0.05) increasing during storage time, and it reached 2.13 mg MDA/^kg^ on the 14th day. However, the treated samples 1.5-L/E/C (0.4 mg MDA/kg) and 2-L/E/C (0.38 mg MDA/kg) remained at relatively low values at the end of the storage period as compared to the control and even to (BHT) samples (0.42 mg MDA/kg) (Table 3). It has been reported that TBARS values lower than 0.40 mg MDA/kg reflected the freshness of the meat and could be admissible to consumers [77]. The low lipid oxidation markers’ values could be attributed to the antioxidant potential of the tested bioactive compounds, which could prevent either free radical generation and the formation of reactive oxygen species or scavenger free radicals and chelate pro-oxidants [78].

Concerning carbonyl content, it has been noticed that it significantly increased (*p* < 0.05) in the control sample from 0.24 to 1.26 μmol/mg during the 14 days of chicken meat storage at refrigerated conditions. Nevertheless, it has been observed that adding L/E/C mixture in treated samples decreased carbonyl formation until the end of the study period. Interestingly, the 2-L/E/C sample (0.69 μmol/mg) was noticed to be more effective than the (BHT) one (0.82 μmol/mg) (Table 3). The formation of the carbonyl compounds indicated the initiation of protein oxidation. The increase in carbonyl content could be the proof of an oxidative deterioration of amino acids, mainly lysine, proline, arginine, and histidine, leading to the alteration of the functionality and the quality of meat proteins [79]. A previous study reported that the use of *Ziziphora clinopodioides* EO (at 0.3%) in chicken meatballs could successfully prevent lipid/protein oxidation owing to its richness in oxygenated monoterpenes [80].

#### 3.3.3. Sensory Evaluation

On day 0 of storage, no significant differences (*p* > 0.05) were perceived in appearance, color, odor, and overall acceptability among all the samples (Table 4). However, the score of all sensory traits significantly (*p* < 0.05) decreased in the control sample until reaching 4.96 (score < 5) on the 10th day, which indicated that the sample became inappropriate for human consumption. These results could be explained by the occurrence of various physiochemical changes related to lipid and protein oxidation reactions, caused essentially by microbial growth and characterized by the appearance of a rancid odor and an unlikable color [65]. However, BHT and the treated samples have given acceptable scores up to 14 days. Hence, it is important to mention that the 2-L/E/C sample represented the best sensory quality and was able to maintain plausible sensory traits throughout the storage period. These findings could be attributed to the appreciable smell and the colorlessness of the tested valuable compounds, which are usually used as aroma and flavor agents in food industries.

#### 3.3.4. Principal Component Analysis (PCA)

**Figure 7:**
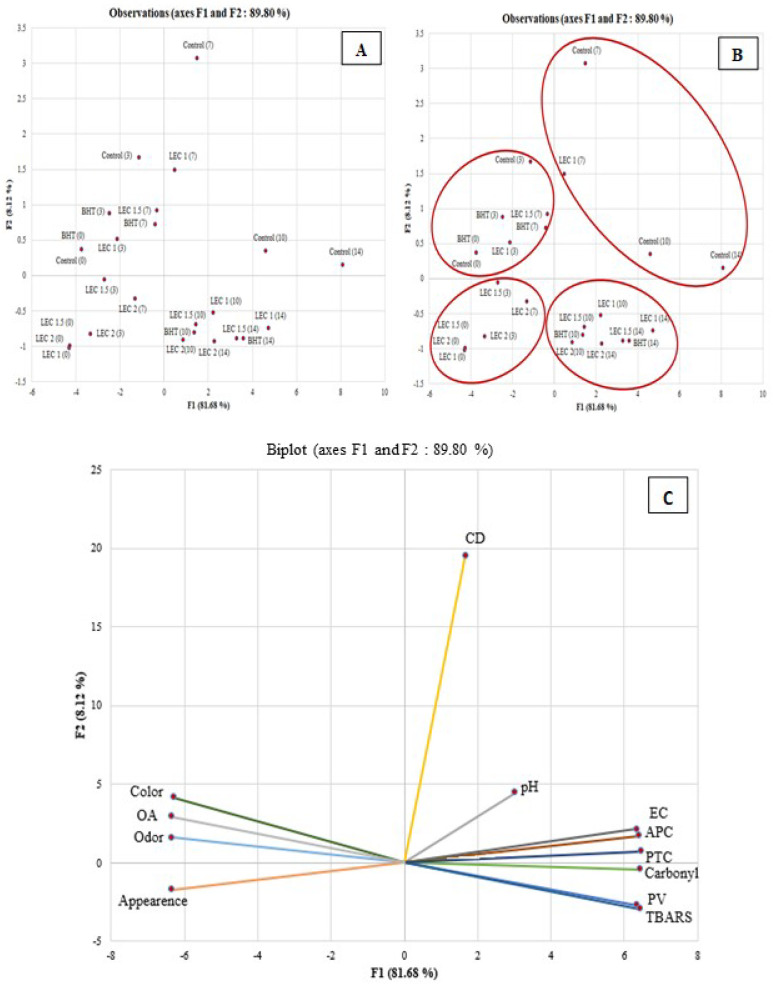
Principal component analysis representing the distribution of the different samples and storage days (**A**,**B**) and the microbial, physicochemical, and descriptive sensory parameter loadings (**C**).

To better understand the impact of L/E/C mixture on raw minced chicken breast meat, lipid/protein oxidation, microbial growth, and sensory parameters were subjected to PCA. Figure 7A–C represent a biplot of the PCA loadings for the two components: F1 and F2. The F1 component predicted 81.68% of the variation, and the F2 brought an additional value of 8.12% to better explain the variation of the studied samples. CD, pH, EC, APC, PTC, color, overall acceptability, and odor had a positive loading on F1 and were found to be opposite to sensory attributes (except the appearance). On the other side, Carbonyl, TBARS, and PV had a negative loading on F1. In addition, all the sensory attributes showed negative loading on F2. The results indicated the presence of a high correlation between protein oxidation (carbonyls), lipid oxidation (TBARS and PV) parameters, and microbiological characteristics (PTC, APC, and Enterobacteriaceae counts), which emphasized the existence of proportional interaction between lipid/protein oxidation and microbial proliferation (Figure 7A–C). Likewise, it has been noticed that the samples tended to deposit towards the right side of the PCA when the storage time was extended. This fact could be explained by the accumulation of primary and secondary lipid and protein oxidation metabolites due to the synergistic activity of pathogenic bacterial lipolytic enzymes and endogenous meat enzymes [81]. Additionally, a positive correlation was observed between control, BHT, 1-L/E/C, and 1.5-L/E/C samples at the beginning of the storage period (Figure 7A–C).

Therefore, the use of 2-L/E/C could prevent protein/lipid oxidation, retards chicken meat spoilage provoked by bacterial growth, and enhances sensory attributes until the end of the refrigerated storage time. In fact, the presence of linalool, eucalyptol, and camphor in mixture could provide synergistic effects, leading to the amelioration of their antioxidant and antibacterial activities [82]. Concisely, these valuable compounds could be a promising and effective alternative to prolong the shelf life of minced chicken breast meat.

## 4. Conclusions

The current research paper focused on the evaluation of the antibacterial activities of L, E, and C against four foodborne bacteria: *S. aureus*, *L. monocytogenes*, *S. enterica* Typhimurium, and *E. coli*. Using SCMD, the obtained results indicated that the optimal predicted mixture displayed the highest antibacterial activity at percentages of 33.5, 33.2, and 33.4% of L, E, and C, respectively. Furthermore, molecular docking simulations indicated that the tested monoterpenes demonstrate effective antibacterial activities by inhibiting pathogenic bacteria virulence factors. Additionally, the in vivo application of the optimized mixture at different concentrations demonstrated that 2-L/E/C at 32 µg/mL exhibited a potential preservative effect, as compared to BHT, by significantly (*p* < 0.05) impeding the proliferation of spoilage microorganisms, delaying lipid/protein oxidation, and ameliorating the sensory quality of minced chicken breast meat samples stored in a refrigerated condition over 14 days. Finally, PCA analysis revealed the presence of a tough link between microbiological and oxidative parameters, as well as sensory attributes.

This paper provided new insights into the identification of the molecular mechanisms of L, E, and C by underlying their ability to control unwanted pathogenic microorganisms. This study revealed their synergetic effect when formulated together to be further implicated as natural preservatives in the extension of the shelf life of chicken products. However, it is necessary to mention that, importantly, the design of innovative and appropriate systems of delivery for these valuable bioactive compounds is needed to preserve their stability in various food matrices.

## Figures and Tables

**Table 1:** Molecular docking results and interaction details of the tested molecules against the targeted protein receptors.

Bacteria	Targets (Receptors)	Compounds (Ligands)	Free Energy of Binding (Kcal/mol)	Number of Interacting Residues	H-Bond Residues
*S. aureus* NCTC 8325	Sortase A	Linalool	−6.5	6	PRO333
		Eucalyptol	−6.3	6	-
		Camphor	−6.3	4	-
*S. enterica* Typhimurium ATCC 700720	YcbB	Linalool	−6.2	7	ASP231
		Eucalyptol	−5.9	3	-
		Camphor	−5.9	6	-
*L. monocytogenes* strain 10403S	Listeriolysin O	Linalool	−6.1	6	TYR414
		Eucalyptol	−5.6	3	-
		Camphor	−5.6	5	-
*E. coli* (strain K12)	PPK	Linalool	−6.7	8	LEU467
		Eucalyptol	−6.6	5	-
		Camphor	−6.5	3	-

**Table 2:** Effects of L/E/C mixture on the microbial load of APC, PTC, and EC (log CFU/g).

Days of Storage						
	Samples	0	3	7	10	14
APC	Control	3.96 ± 0.05 ^aA^	4.82 ± 0.17 ^eB^	5.26 ± 0.13 ^cC^	6.48 ± 0.22 ^cD^	7.67 ± 0.25 ^eE^
	BHT	3.96 ± 0.05 ^aA^	4.46 ± 0.12 ^dB^	4.96 ± 0.12 ^bC^	5.08 ± 0.19 ^aC^	5.68 ± 0.17 ^bD^
	1-L/E/C	3.96 ± 0.05 ^aA^	4.35 ± 0.15 ^cB^	4.99 ± 0.09 ^bC^	5.43 ± 0.15 ^bD^	6.12 ± 0.21 ^dE^
	1.5-L/E/C	3.96 ± 0.05 ^aA^	4.27 ± 0.1 ^bB^	4.87 ± 0.07 ^aC^	5.04 ± 0.16 ^aD^	5.91 ± 0.19 ^cE^
	2-L/E/C	3.96 ± 0.05 ^aA^	4.05 ± 0.09 ^aA^	4.82 ± 0.11 ^aB^	4.94 ± 0.11 ^aB^	5.62 ± 0.12 ^aC^
PTC	Control	3.46 ± 0.02 ^aA^	4.26 ± 0.13 ^cB^	5.11 ± 0.15 ^cC^	6.30 ± 0.19 ^cD^	7.51 ± 0.23 ^eE^
	BHT	3.46 ± 0.02 ^aA^	3.89 ± 0.1 ^bB^	4.91 ± 0.12 ^bC^	4.98 ± 0.12 ^aC^	5.61 ± 0.11 ^bD^
	1-L/E/C	3.46 ± 0.02 ^aA^	3.96 ± 0.06 ^bcB^	4.94 ± 0.07 ^bC^	5.38 ± 0.18 ^bD^	5.96 ± 0.13 ^dE^
	1.5-L/E/C	3.46 ± 0.02 ^aA^	3.84 ± 0.08 ^bB^	4.88 ± 0.09 ^abC^	4.95 ± 0.1 ^aC^	5.78 ± 0.15 ^cD^
	2-L/E/C	3.46 ± 0.02 ^aA^	3.78 ± 0.11 ^aA^	4.73 ± 0.08 ^aB^	4.92 ± 0.09 ^aB^	5.49 ± 0.1 ^aC^
EC	Control	<1	1.45 ± 0.03 ^dA^	1.954 ± 0.02 ^dB^	2.14 ± 0.01 ^dC^	3.20 ± 0.11 ^cD^
	BHT	<1	1.20 ± 0.025 ^cA^	1.31 ± 0.07 ^bA^	1.53 ± 0.02 ^bB^	1.98 ± 0.04 ^aC^
	1-L/E/C	<1	1.23 ± 0.027 ^cA^	1.49 ± 0.04 ^cB^	1.86 ± 0.025 ^cC^	2.06 ± 0.06 ^bD^
	1.5-L/E/C	<1	1.12 ± 0.01 ^bA^	1.27 ± 0.03 ^bB^	1.50 ± 0.03 ^bC^	1.96 ± 0.03 ^aD^
	2-L/E/C	<1	1.02 ± 0.02 ^aA^	1.14 ± 0.01 ^aB^	1.43 ± 0.01 ^aC^	1.90 ± 0.02 ^aD^

APC: aerobic plate counts; PTC: psychotropic count; EC: Enterobacteriaceae; ±, standard deviation (SD) of three replicates; a–e: mean values within all the samples not followed by a similar letter in the same column vary significantly (*p* < 0.05); A–E: mean values during storage not followed by a similar letter in the same line vary significantly (*p* < 0.05).

**Table 3:** Effects of L/E/C treatment on pH, PV, CD, TBARS, and carbonyl contents.

	Days of Storage					
	Samples	0	3	7	10	14
pH	Control	5.79 ± 0.12 ^aA^	6.05 ± 0.15 ^bB^	6.24 ± 0.13 ^bC^	6.51 ± 0.16 ^cD^	6.84 ± 0.17 ^cE^
	BHT	5.79 ± 0.12 ^aA^	5.95 ± 0.17 ^aB^	5.97 ± 0.19 ^aB^	6.03 ± 0.17 ^aB^	6.2 ± 0.13 ^bC^
	1-L/E/C	5.79 ± 0.12 ^aA^	6.03 ± 0.24 ^bB^	6.11 ± 0.26 ^bB^	6.21 ± 0.14 ^bC^	6.31 ± 0.14 ^bD^
	1.5-L/E/C	5.79 ± 0.12 ^aA^	5.99 ± 0.32 ^aB^	6.01 ± 0.15 ^aB^	6.15 ± 0.13 ^bC^	6.21 ± 0.12 ^bC^
	2-L/E/C	5.79 ± 0.12 ^aA^	5.87 ± 0.12 ^aA^	5.99 ± 0.14 ^aB^	6.09 ± 0.11 ^aC^	6.14 ± 0.14 ^aC^
PV	Control	0.04 ± 0.001 ^aA^	0.12 ± 0.004 ^cB^	0.32 ± 0.01 ^dC^	0.47 ± 0.02 ^cD^	0.85 ± 0.02 ^dE^
	BHT	0.04 ± 0.001 ^aA^	0.07 ± 0.001 ^aB^	0.24 ± 0.002 ^bC^	0.31 ± 0.009 ^bD^	0.6 ± 0.02 ^bE^
	1-L/E/C	0.04 ± 0.001 ^aA^	0.09 ± 0.001 ^bB^	0.28 ± 0.001 ^cC^	0.38 ± 0.008 ^bD^	0.71 ± 0.01 ^cE^
	1.5-L/E/C	0.04 ± 0.001 ^aA^	0.08 ± 0.001 ^bB^	0.23 ± 0.002 ^bC^	0.34 ± 0.002 ^bD^	0.65 ± 0.02 ^bE^
	2-L/E/C	0.04 ± 0.001 ^aA^	0.06 ± 0.001 ^aA^	0.19 ± 0.002 ^aB^	0.28 ± 0.001 ^aC^	0.43 ± 0.01 ^aD^
CD	Control	0.51 ± 0.02 ^aA^	1.27 ± 0.01 ^eC^	1.89 ± 0.014 ^dD^	0.85 ± 0.021 ^dB^	0.76 ± 0.025 ^cB^
	BHT	0.51 ± 0.02 ^aA^	0.97 ± 0.014 ^dB^	0.99 ± 0.013 ^bB^	0.57 ± 0.02 ^bA^	0.51 ± 0.018 ^bA^
	1-L/E/C	0.51 ± 0.02 ^aA^	0.82 ± 0.014 ^cC^	1.28 ± 0.04 ^cD^	0.66 ± 0.02 ^cB^	0.61 ± 0.01 ^cB^
	1.5-L/E/C	0.51 ± 0.02 ^aA^	0.69 ± 0.012 ^bB^	1.07 ± 0.01 ^bC^	0.58 ± 0.01 ^bB^	0.54 ± 0.02 ^bA^
	2-L/E/C	0.51 ± 0.02 ^aA^	0.59 ± 0.01 ^aA^	0.67 ± 0.02 ^aC^	0.49 ± 0.02 ^aB^	0.45 ± 0.01 ^aB^
TBARS	Control	0.1 ± 0.004 ^aA^	0.32 ± 0.01 ^dB^	0.89 ± 0.09 ^dC^	1.65 ± 0.03 ^cD^	2.13 ± 0.07 ^cE^
	BHT	0.1 ± 0.004 ^aA^	0.21 ± 0.011 ^bB^	0.28 ± 0.042 ^bB^	0.36 ± 0.004 ^aC^	0.42 ± 0.03 ^aD^
	1-L/E/C	0.1 ± 0.004 ^aA^	0.28 ± 0.01 ^cB^	0.56 ± 0.011 ^cC^	0.76 ± 0.03 ^bD^	1.12 ± 0.05 ^bE^
	1.5-L/E/C	0.1 ± 0.004 ^aA^	0.24 ± 0.011 ^bB^	0.30 ± 0.012 ^bC^	0.34 ± 0.02 ^aC^	0.4 ± 0.05 ^aD^
	2-L/E/C	0.1 ± 0.004 ^aA^	0.14 ± 0.012 ^aA^	0.22 ± 0.01 ^aB^	0.30 ± 0.01 ^aC^	0.38 ± 0.04 ^aD^
Carbonyls	Control	0.24 ± 0.008 ^aA^	0.52 ± 0.02 ^dB^	0.68 ± 0.018 ^cC^	0.89 ± 0.03 ^dD^	1.26 ± 0.014 ^dE^
	BHT	0.24 ± 0.008 ^aA^	0.43 ± 0.012 ^cB^	0.45 ± 0.013 ^aB^	0.56 ± 0.02 ^aC^	0.82 ± 0.03 ^bD^
	1-L/E/C	0.24 ± 0.008 ^aA^	0.48 ± 0.01 ^cB^	0.57 ± 0.013 ^bC^	0.78 ± 0.02 ^cD^	1.12 ± 0.04 ^cE^
	1.5-L/E/C	0.24 ± 0.008 ^aA^	0.36 ± 0.011 ^bB^	0.49 ± 0.014 ^aC^	0.73 ± 0.01 ^cD^	0.89 ± 0.03 ^bE^
	2-L/E/C	0.24 ± 0.008 ^aA^	0.29 ± 0.01 ^aA^	0.43 ± 0.012 ^aB^	0.65 ± 0.01 ^bC^	0.69 ± 0.02 ^aC^

±: standard deviation (SD) of three replicates; a–e: mean values within all the samples not followed by a similar letter in the same column vary significantly (*p* < 0.05); A–E: mean values during storage not followed by a similar letter in the same line vary significantly (*p* < 0.05).

**Table 4:** Effects of L/E/C mixture on the appearance, color, odor, and OA of raw minced chicken breast samples.

Days of Storage						
	Samples	0	3	7	10	14
Appearance	Control	8.6 ± 0.24 ^aE^	6.7 ± 0.21 ^aD^	5.58 ± 0.2 ^aC^	4.96 ± 0.12 ^aB^	4.21 ± 0.08 ^aA^
	BHT	8.6 ± 0.24 ^aD^	7.5 ± 0.17 ^bC^	6.72 ± 0.05 ^bB^	6.48 ± 0.21 ^bB^	5.86 ± 0.17 ^cA^
	1-L/E/C	8.6 ± 0.24 ^aD^	7.2 ± 0.14 ^bD^	6.68 ± 0.23 ^bC^	6.41 ± 0.19 ^bB^	5.36 ± 0.04 ^bA^
	1.5-L/E/C	8.6 ± 0.24 ^aC^	7.5 ± 0.23 ^bB^	6.78 ± 0.19 ^dA^	6.72 ± 0.14 ^bA^	6.58 ± 0.13 ^bA^
	2-L/E/C	8.6 ± 0.24 ^aD^	7.79 ± 0.29 ^cC^	7.6 ± 0.11 ^bC^	7.08 ± 0.18^cB^	5.96 ± 0.11^cA^
Color	Control	8.25 ± 0.19 ^aE^	7.23 ± 0.09 ^aD^	6.89 ± 0.26 ^bC^	5.13 ± 0.06 ^aB^	4.08 ± 0.06 ^aA^
	BHT	8.25 ± 0.19 ^aD^	7.61 ± 0.07 ^bC^	6.98 ± 0.18 ^cB^	5.32 ± 0.14 ^cA^	5.21 ± 0.12 ^cA^
	1-L/E/C	8.25 ± 0.19 ^aE^	7.43 ± 0.16 ^cD^	6.66 ± 0.08 ^aC^	5.27 ± 0.17 ^bB^	4.98 ± 0.09 ^bA^
	1.5-L/E/C	8.25 ± 0.19 ^aD^	7.58 ± 0.25 ^cC^	6.83 ± 0.19 ^bB^	5.36 ± 0.09 ^cA^	5.17 ± 0.13 ^cA^
	2-L/E/C	8.25 ± 0.19 ^aD^	7.62 ± 0.22 ^bC^	7.12 ± 0.22 ^dB^	5.47 ± 0.18 ^dA^	5.38 ± 0.14 ^dA^
Odor	Control	8.02 ± 0.16 ^aD^	7.25 ± 0.17 ^aC^	5.08 ± 0.06 ^aB^	4.86 ± 0.05 ^aB^	4.23 ± 0.1 ^aA^
	BHT	8.02 ± 0.16 ^aD^	7.65 ± 0.25 ^cC^	6.87 ± 0.12 ^cB^	5.76 ± 0.13 ^cA^	5.49 ± 0.17 ^cA^
	1-L/E/C	8.02 ± 0.16 ^aD^	7.53 ± 0.27 ^bC^	6.41 ± 0.03 ^bB^	5.47 ± 0.07 ^bA^	5.31 ± 0.06 ^bA^
	1.5-L/E/C	8.02 ± 0.16 ^aD^	7.68 ± 0.10 ^cC^	6.81 ± 0.17 ^cB^	5.69 ± 0.17 ^cA^	5.45 ± 0.18 ^cA^
	2-L/E/C	8.02 ± 0.16 ^aD^	7.84 ± 0.29 ^dC^	6.96 ± 0.12 ^dB^	5.94 ± 0.15 ^dA^	5.72 ± 0.15 ^dA^
Overall acceptability	Control	8.06 ± 0.11 ^aE^	7.23 ± 0.20 ^aD^	6.33 ± 0.09 ^aC^	5.32 ± 0.08 ^aB^	4.63 ± 0.08 ^aA^
	BHT	8.06 ± 0.11 ^aD^	7.52 ± 0.13 ^cC^	6.71 ± 0.14 ^cB^	5.58 ± 0.16 ^bBA^	5.12 ± 0.11 ^cA^
	1-L/E/C	8.06 ± 0.11 ^aE^	7.46 ± 0.15 ^bD^	6.58 ± 0.11 ^bC^	5.48 ± 0.12 ^bB^	4.96 ± 0.07 ^bA^
	1.5-L/E/C	8.06 ± 0.11 ^aD^	7.59 ± 0.21 ^cC^	6.75 ± 0.23 ^cB^	5.67 ± 0.12 ^cBA^	5.23 ± 0.12 ^dA^
	2-L/E/C	8.06 ± 0.11 ^aD^	7.73 ± 0.08 ^dC^	6.86 ± 0.15 ^dB^	5.79 ± 0.07 ^cA^	5.47 ± 0.15 ^eA^

±, standard deviation (SD) of three replicates; a–e: mean values within all the samples not followed by a similar letter in the same column vary significantly (*p* < 0.05); A–E: mean values during storage not followed by a similar letter in the same line vary significantly (*p* < 0.05).

## Data Availability

The original contributions presented in the study are included in the article/Supplementary Materials, further inquiries can be directed to the corresponding author.

## Supplementary Materials

The following supporting information can be downloaded at https://www.mdpi.com/article/10.3390/foods13060965/s1. Table S1: Experimental variables and responses used in the mixture design; Table S2: ANOVA results of regression models from mixture design for MICs optimization.
