# Supplementary material for: Disclosing the Functional Potency of Three Oxygenated Monoterpenes in Combating Microbial Pathogenesis: From Targeting Virulence Factors to Chicken Meat Preservation
Source: Foods. 2024 Mar 21;13(6):965. doi: 10.3390/foods13060965 (PMC10970324; doi:10.3390/foods13060965)
Supplement: Supplementary file 1 [file foods-13-00965-s001.zip › foods-2915862-supplementary.pdf]

Supplementary Table S1. Experimental variables and responses used in the mixture design.

	Variables			MIC *S. aureus* (μg/mL)		MIC *S. enterica* (μg/mL)		MIC *E. coli* (μg/mL)		MIC *L. monocytogenes* (μg/mL)	
**Runs**	**Linalool (A)**	**Eucalyptol (B)**	**Camphor (C)**	**Exp.**	**Pred.**	**Exp.**	**Pred.**	**Exp.**	**Pred.**	**Exp.**	**Pred.**
**1**	1.000	0.000	0.000	168±6.765^{d}	168.40	168±8.25^{d}	173.40	168±7.84^{c}	186.60	168±7.755^{e}	155.70
**2**	0.000	1.000	0.000	168±6.765^{d}	195.90	168±8.25^{d}	187.60	168±7.84^{c}	197.80	168±7.755^{e}	159.50
**3**	0.000	0.000	1.000	168±6.765^{d}	161.00	168±8.25^{d}	162.20	168±7.00^{c}	181.40	168±7.755^{e}	176.10
**4**	0.500	0.500	0.000	42±1.681^{b}	34.275	42±2.05^{b}	40.97	84±3.28^{b}	74.40	84±3.854^{d}	76.20
**5**	0.500	0.000	0.500	84±3.362^{c}	93.325	10.5±0.50^{a}	15.60	42±1.64^{a}	49.02	84±3.854^{d}	83.82
**6**	0.000	0.500	0.500	84±3.362^{c}	77.90	42±2.05^{b}	39.77	42±1.64^{a}	40.22	42±1.927^{c}	46.57
**7**	0.333	0.333	0.333	21±0.861^{a}	23.34	42±2.05^{b}	49.27	42±1.64^{a}	38.90	10.5±0.470^{a}	13.33
**8**	0.6667	0.1667	0.1667	42±1.681^{b}	55.54	84±4.10^{c}	78.10	42±1.64^{a}	41.02	21±0.987^{b}	29.60
**9**	0.1667	0.6667	0.1667	42±1.681^{b}	55.44	42±2.05^{b}	37.55	84±3.28^{b}	78.33	21±0.987^{b}	28.44
**10**	0.1667	0.1667	0.6667	42±1.681^{b}	49.80	42±2.05^{b}	40.63	84±3.28^{b}	87.51	84±3.854^{d}	79.52

Exp: Experimental, Pred: Predicted. For each run, responses with different letters are significantly different (one-way analysis of variance, $p < 0.05$; Tukey's test), ± standard deviation (SD) of three measurements.

Supplementary Table S2. ANOVA results of regression models from mixture design for MICs optimization

Source	DF	Seq SS	Adj SS	Adj MS	F-value	*p*-value
S. aureus						
**Regression**	6	33809.2	33809.2	5634.9	5.93	0.086
**Linear**	2	446.3	744.9	372.5	0.39	0.706
**Quadratic**	3	33354.7	21038.4	7012.8	7.38	0.067
**A × B**	1	17760.7	14773.8	14773.8	15.55	0.029
**A × C**	1	7173.6	5884.5	5884.5	6.19	0.089
**B × C**	1	8420.3	6831.3	6831.3	7.19	0.075
**Special cubic**	1	8.2	8.2	8.2	0.01	0.932
**A× B × C**	1	8.2	8.2	8.2	0.01	0.932
**Residual Error**	3	2850.9	2850.9	950.3		
**Total**	9	36660.1				
S. enterica						
**Regression**	6	37765.8	37765.8	6294.3	10.51	0.040
**Linear**	2	724.1	355.5	177.7	0.30	0.763
**Quadratic**	3	34211.1	32108.2	10702.7	17.87	0.020
**A × B**	1	10103.9	13154.3	13154.3	21.97	0.018
**A × C**	1	12682.2	15654.2	15654.2	26.14	0.014
**B × C**	1	11425	14229.7	14229.7	23.76	0.016
**Special cubic**	1	2830.7	2830.7	2830.7	4.73	0.118
**A × B × C**	1	2830.7	2830.7	2830.7	4.73	0.118
**Residual Error**	3	1796.6	1796.6	598.9		
**Total**	9	39562.4				
E. coli						
**Regression**	6	35114.3	35114.3	5852.4	11.83	0.034
**Linear**	2	434.8	153.9	76.9	0.16	0.862
**Quadratic**	3	34429.6	24644.8	8214.9	16.6	0.023
**A × B**	1	9525	9375.2	9375.2	18.95	0.022
**A × C**	1	12939	12309.8	12309.8	24.88	0.015
**B × C**	1	11965.6	11311.1	11311.1	22.86	0.017

**Special cubic**	1	249.9	249.9	249.2	0.51	0.529
**A × B × C**	1	249.9	249.9	249.9	0.51	0.529
**Residual Error**	3	1484.6	1484.6	494.9		
**Total**	9	36598.9				
L. monocytogenes						
**Regression**	6	32059.3	32059.3	5343.22	5.54	0.094
**Linear**	2	322.3	258.6	129.31	0.13	0.880
**Quadratic**	3	30903	15175	5058.33	5.24	0.103
**A × B**	1	8638.8	5521.6	5521.57	5.72	0.097
**A × C**	1	7392.9	4552.2	4552.15	4.72	0.118
**B × C**	1	14871.2	9928.2	9928.15	10.29	0.049
**Special cubic**	1	834	834	834.01	0.86	0.421
**A × B × C**	1	834	834	834.01	0.86	0.421
**Residual Error**	3	2895.1	2895.1	965.02		
**Total**	9	34954.4				
